# Investigating the Impact of Delivery System Design on the Efficacy of Self-Amplifying RNA Vaccines

**DOI:** 10.3390/vaccines8020212

**Published:** 2020-05-08

**Authors:** Giulia Anderluzzi, Gustavo Lou, Simona Gallorini, Michela Brazzoli, Russell Johnson, Derek T. O’Hagan, Barbara C. Baudner, Yvonne Perrie

**Affiliations:** 1 Strathclyde Institute of Pharmacy and Biomedical Sciences, University of Strathclyde, Glasgow G4 ORE, Scotland, UK; giulia.anderluzzi@strath.ac.uk (G.A.); gustavo.lou-ramirez@strath.ac.uk (G.L.); 2 GSK, 53100 Siena 1, Italy; simona.x.gallorini@gsk.com (S.G.); michela.x.brazzoli@gsk.com (M.B.); barbara.c.baudner@gsk.com (B.C.B.); 3 GSK, Rockville, MD 9911, USA; russell.n.johnson@gsk.com (R.J.);

**Keywords:** self-amplifying RNA, liposomes, polymeric nanoparticles, solid lipid nanoparticles, emulsions, antigen expression, immunogenicity

## Abstract

messenger RNA (mRNA)-based vaccines combine the positive attributes of both live-attenuated and subunit vaccines. In order for these to be applied for clinical use, they require to be formulated with delivery systems. However, there are limited in vivo studies which compare different delivery platforms. Therefore, we have compared four different cationic platforms: (1) liposomes, (2) solid lipid nanoparticles (SLNs), (3) polymeric nanoparticles (NPs) and (4) emulsions, to deliver a self-amplifying mRNA (SAM) vaccine. All formulations contained either the non-ionizable cationic lipid 1,2-dioleoyl-3-trimethylammonium-propane (DOTAP) or dimethyldioctadecylammonium bromide (DDA) and they were characterized in terms of physico-chemical attributes*,* in vitro transfection efficiency and in vivo vaccine potency. Our results showed that SAM encapsulating DOTAP polymeric nanoparticles, DOTAP liposomes and DDA liposomes induced the highest antigen expression in vitro and, from these, DOTAP polymeric nanoparticles were the most potent in triggering humoral and cellular immunity among candidates in vivo.

## 1. Introduction

Messenger RNA (mRNA)-based vaccines have emerged as a transformative approach, due to their ability to elicit both humoral and cellular immunity [1,2,3,4]. Recently, there has been an increased interest in applying this technology by numerous companies, including Biomay AG, BioNTech AG, CureVac, Ethris, eTheRNA BVBA, Moderna Therapeutics, Novartis Vaccines, Ribological GmbH, Tekmira Pharmaceuticals Corporation and TriLink BioTherapeutics [5]. In contrast to plasmid DNA (pDNA) vaccines, mRNA does not need to be transported across the nuclear membrane and it can directly induce antigen expression in the cytosol, such that any potential genomic integration is avoided [6]. Moreover, mRNA vaccines induce transient antigen expression, while DNA vaccines provide a long-lasting expression, thus mimicking an acute viral infection. Although a transitory gene expression might be desirable as it minimizes potential risks of genetic transformation, this inevitably affects mRNA potency, thus necessitating a dose increase [7]. One strategy to augment mRNA performance is to include genetic elements that enable self-amplification. Here, the self-amplifying mRNA (SAM) is based on an alphavirus genome having a positive single stranded RNA (+ssRNA). Importantly, the structural protein genes of respective alphavirus that enable the formation of infectious viral particles have been substituted with genes encoding the antigen of interest [8]. However, the virus amplification machinery (i.e., polyproteins involved in the replication of the RNA in multiple copies) have been maintained, hence allowing SAM vaccines to self-amplify over time and consequently inducing more potent immune responses compared to conventional non-amplifying mRNA vaccines [9,10,11]. 

As with other nucleic acid-based technologies, SAM is prone to enzymatic degradation once injected into the body [12]. Moreover, due to the intrinsic hydrophilic nature and high molecular weight of SAM, crossing the cell membrane and traversing the endosome for effective delivery to the cytoplasm presents a formidable challenge. Viral vectors have been used to efficiently deliver nucleic acid into cell cytoplasm [13,14]; however, because of pre-existing or vaccine-induced antivector immunity, vaccine potency might be reduced after repeated vaccinations. Physical methods such as gene gun and electroporation (EP) can also be used to deliver mRNA, although, these techniques are difficult to scale, and devices are bulky and can be cost-prohibitive for mass vaccine strategies [15,16]. Considering these obstacles, employing a synthetic delivery vehicle for the nucleic acid is advantageous. Many studies have reported enhanced immunogenicity when SAM vaccines are delivered by a particulate delivery system [17,18,19]. Of the systems tested, lipid nanoparticles (LNPs) are the most advanced in enabling the clinical potential of genetic drugs, such as small interfering RNA (siRNA) and mRNA, with several siRNA-LNP systems already in clinical trials as antiviral, anticancer and genetic disorder therapies [20]. Generally, LNPs are composed of a complexing amino lipid (either ionizable or non-ionizable), a phospholipid, cholesterol, a poly (ethylene glycol)-lipid conjugate and the therapeutic nucleic acid [21]. The choice of complexing lipid is a key element which strongly dictates LNP potency. The ionizable amino lipids heptatriaconta-6,9,28,31-tetraen-19-yl 4-(dimethylamino) butanoate (DLin-MC3-DMA) and 2,2-dilinoleyl-4-(2-dimethylaminoethyl)-[1,3]-dioxolane (DLin-KC2-DMA) have low toxicity profiles and are among the most promising. An siRNA-LNP formulation using DLin-MC3-DMA was recently approved by the Food and Drug administration (FDA) for the treatment of polyneuropathy of hereditary transthyretin-mediated amyloidosis in adults [5]. However, the application of LNPs based on DLin- and other ionizable lipids to deliver nucleic acids may be limited by their high cost. Hence, developing more commercially competitive carriers with comparable delivery profiles (in terms of SAM protection and antigen expression) is of great interest. There is a wide range of nanoparticles that may offer advantages in terms of SAM delivery. Here, we have investigated four non-viral vectors for the delivery of SAM: (1) liposomes, (2) solid lipid nanoparticles (SLNs), (3) polymeric nanoparticles (NPs) and (4) emulsions. All particles were formulated containing either the commercially available non-ionizable cationic lipid 1,2-dioleoyl-3-trimethylammonium-propane (DOTAP) or dimethyldioctadecylammonium bromide (DDA), as they have been extensively investigated as transfection agents or immunostimulants respectively, for both nucleic acid [22] and subunit vaccines [23]. Furthermore, their safety profile is well-documented in numerous clinical trials [24,25,26]. While liposomes, oil-in-water emulsions and polymeric NPs have been widely reported for the delivery of nucleic acids [27,28,29,30,31], there are limited publications on the in vivo delivery of DNA or mRNA using SLNs. Moreover, to the best of our knowledge, a head-to-head comparison of these four different carriers for the delivery of SAM has not been undertaken.

To formulate these lipid-based nanovectors, we applied scalable manufacturing methods (microfluidic mixers or microfluidization). Microfluidic mixers are relatively simple and efficient systems, capable of producing synthetic particles with consistent size and biophysical properties, including encapsulation of nucleic acids due to reliable laminar flow dynamics [32,33,34]. To prepare emulsions, the microfluidization process was used. This method is versatile and has been employed for the manufacturing of different kinds of formulations [35] for large-scale production. By adjusting process parameters, a tight control over particle characteristics can be achieved. When considering the lipid composition to use, LNPs previously evaluated for intramuscular (i.m.) mRNA vaccine delivery were those originally developed for intravenous (i.v.) delivery of siRNA to the liver [10,36]. However, a recent investigation suggested that the optimal composition of ionizable LNPs is dictated by the pKa of the ionizable lipid and is route-dependent; specifically, maximum LNP potency after i.v. injection was achieved using ionizable lipids with a pKa of 6.2 to 6.4, whilst an optimal pKa range from 6.6 to 6.8 was suggested for i.m. delivery [37]. However, in a separate study which evaluated the immunogenicity of a SAM vaccine formulated in LNPs composed of either cationic or ionizable lipids, it was observed that all LNPs induced equivalent humoral immune responses, indicating that the use of ionizable lipids is not necessarily a pre-requisite [38]. Therefore, further consideration of SAM formulation components that offer a lower cost of goods could be advantageous. In addition, considering the findings of Blakney and co-workers [38], in which LNPs formulated with SAM either on the interior or exterior of the particles were equally able to protect SAM from enzymatic degradation and induce comparable humoral immune responses, herein, formulations were prepared with SAM either encapsulated or adsorbed. To investigate these factors, a SAM encoding rabies virus glycoprotein G (SAM-RVG) was used and the immunogenicity was compared to that of the licensed rabies vaccine Rabipur and the GlaxoSmithKline (GSK, Rockville, MD, USA) proprietary cationic nanoemulsion (CNE56) as benchmarks for the rabies vaccine and mRNA delivery, respectively.

## 2. Materials and Methods 

### 2.1. Materials 

Poly (D, L-lactide-co-glycolide) lactide: glycolide (50:50), mol wt 30,000–60,000, Dimethyl Sulfoxide (DMSO), Tristearin (Grade II-S, ≥90%), cholesterol, squalene, penicillin-streptomycin, L-glutamine and Brefeldin A were purchased from Sigma Aldrich Company Ltd., UK. Distearoylphosphatidylcholine (DSPC), 1,2-dimyristoyl-rac-glycero-3-methoxypolyethylene glycol-2000 (DMG-PEG2000), Dimethyldioctadecylammonium bromide (DDA) and 1,2-dioleoyl-3-trimethylammonium-propane (DOTAP) were obtained from Avanti Polar Lipids, US. Ethanol, methanol, 1,1’-Dioctadecyl-3,3,3’,3’-Tetramethylindocarbocyanine Perchlorate (DilC), Live/Dead Fixable Aqua Dead Cell Stain, Lipofectamine 2000, Gibco phosphate buffer saline (PBS), Tween 20 and Span 80, RNase A, proteinase K, Northern Max formaldehyde load dye, Northern Max running 10X buffer, Ambion millennium RNA, SYBR gold nucleic acid stain marker (10,000X in DMSO), 3 M sodium acetate buffer pH 5.2, Ribo Green RNA assay kit, opti- Minimal Essential Medium (MEM), Alexa Fluor 488-labeled goat anti-mouse IgG2a Cross-Adsorbed secondary antibody and allophycocyanin (APC) Zenon antibody labeling kit for mouse IgG2a and diethylpyrocarbonate (DEPC)-treated water were purchased from Thermo Fisher, UK. Tris(hydroxymethyl)aminomethane TRIS Ultra-Pure was obtained from Fisher Scientific, UK. Roswell Park Memorial Institute 1640 medium (RPMI-1640), Hank’s balance salt solution (HBSS) trypsin ethylenediaminetetraacetic acid (-EDTA,0.25%), Dulbecco’s Modified Eagle Medium (DMEM) and heat-inactivated fetal calf serum (HI-FCS) were obtained from Gibco, Italy. 10X Perm/Wash buffer and cytofix-cytoperm solution was obtained from BD Biosciences, UK. Anti-CD16/CD32 monoclonal antibody (Fc block) was obtained by BD Biosciences, UK. Anti-CD3-APC, anti-CD28, anti-CD4 BV510, anti-CD107a FITC, anti-IFN-γ Brilliant Violet 785, anti-IL-2 PE-Cy5.5, anti-TNF Brilliant Violet 605, anti-CD44 V450, anti-IL17 PE and anti-CD8 PE-CF594-conjugated monoclonal antibodies were obtained from BD Pharmingen, UK. Mini Ready Agarose precast gels 1% TAE and PLATELIA Rabies II Kit were obtained from Bio-Rad, Italy. Live/dead fixable dead cell stain near-infraredwas purchased from Life Technologies, Italy. The rabies peptide pool G1/G2/G3 was obtained from Genescript, US. Rabipur is a trademark owned by the GSK group of companies. 

### 2.2. SAM Synthesis 

DNA plasmids encoding the self-amplifying RNAs were constructed using standard molecular techniques based on previous work by Geall and co-workers [10]. Plasmids were amplified in Escherichia coli and purified using Qiagen Plasmid Maxi kits (Qiagen, Germantown, MD, USA). DNA was linearized immediately following the 3′ end of the self-amplifying RNA sequence by restriction digest. Linearized DNA templates were transcribed into RNA using the MEGAscript T7 kit (Life Technologies, Carlsbad, CA, MA, USA) and purified by LiCl precipitation. RNA was then capped using the Vaccinia Capping system (New England BioLabs, Ipswich, MA, USA) and purified by LiCl precipitation before formulation. Self-amplifying RNA encoding the rabies virus glycoprotein (SAM-RVG) was used in the study.

### 2.3. Preparation of SAM Formulations 

#### 2.3.1. Formulation of Liposome, Solid-lipid Nanoparticles and Polymeric Nanoparticles

Cationic formulations were prepared using the NanoAssemblr Platform (Precision Nanosystems Inc., Vancouver, BC, Canada) in a staggered herringbone micromixer. Briefly, with respect to liposomes, lipid mixtures composed of 1,2-dioleoyl-sn-glycero-3-phosphoethanolamine (DOPE) and a cationic lipid (DOTAP or DDA) were prepared in methanol at 1:1 w/w. Tris buffer pH 7.4 10 mM was used as aqueous phase. The aqueous: organic flow rate ratio (FRR) and the total flow rate (TFR) were set at 1:1 and 15 mL/min, respectively. Regarding SLNs, a mixture of Tristearin, DOTAP or DDA (1:1 w/w) and 2 mol% of DMG-PEG2000 were dissolved in 1 mL ethanol (70 °C) to allow complete lipids dissolution. Tris buffer pH 7.4 10 mM was used as aqueous phase. Process parameters were fixed at flow rate ratios of 3:1 (aqueous to solvent) and total flow rates of 15 mL/min. Similarly, to prepare NPs, poly (D, L-lactide-co-glycolide) lactide: glycolide (50:50), mol/w 30,000–60,000 (PLGA) and DOTAP or DDA 1:1 w/w were dissolved in DMSO and acetate buffer 100 mM, pH 6 was used as aqueous phase. The FRR and TFR were set up at 1:1 and 15 mL/min. For all formulations, the organic and an aqueous phase were injected simultaneously in the micromixer, particles were collected in a 15 mL falcon tube and dialyzed for 1 hour at room temperature against the same formulation buffer for removal of residual organic solvent (dialysis membrane cut off 14,000 Da).

#### 2.3.2. Formulation of Emulsions

To formulate oil in water (o/w) emulsions, DOTAP or DDA (4.3% w/w) were mixed with squalene (5.0%, w/w) and heated above cationic lipids transition temperature to allow complete dissolution. Then, tween (0.5%, w/w), span (0.5%, w/w) and Tris buffer 10mM pH 7.4 were added and the mixture was vortexed for 1 min to provide a homogeneous feedstock. This primary emulsion was loaded in the inlet reservoir of an Avestin Emulsiflex C3 (AVESTIN, Inc, Ottawa, Canada) and passed 5 times at 25,000 psi to reduce particle size. CNE56 was prepared by using the method previously described by Brito and co-workers [39].

#### 2.3.3. Preparation of SAM-Loaded Formulations 

To encapsulate SAM-RVG inside formulations, liposomes, SLNs and NPs were formulated with the addition of RNA 8:1 mol/mol N:P (N in the cationic lipid and P in SAM) in the aqueous phase prior to micromixing, following the protocol in Section 2.3.1. To load SAM on the surface, the nucleic acid (8:1 mol/mol N:P) was added dropwise into the suspensions of liposomes, SLNs, NPs and emulsions under mild stirring. SAM-adsorbing formulations were allowed to complex at 4 °C for at least 2 hours. Prior to in vitro incubation or in vivo administration, formulations were diluted to dosing concentration.

### 2.4. Quantification of SAM Loading and Adsorption Efficiency 

SAM encapsulation efficiency (EE) was measured using the Quant-iT RiboGreen RNA Assay Kit following the manufacturer’s instructions. Briefly, 100 µL of the diluted fluorescent dye was added to each formulation and incubated in absence of light for 5 min. This allowed the dye to quantitatively bind free nucleic acid. The concentration of non-loaded SAM was determined by measuring fluorescence (λ_em_ = 480 nm, λ_ex_ = 520nm) using a Synergy H1 Hybrid Multi-Mode Reader (BioTek Instruments Inc, Winooski, VT, USA). The actual loading was obtained by subtracting the unloaded SAM to the initial nucleic acid concentration. Furthermore, to quantify SAM adsorption yield, SAM adsorbing samples were ultracentrifuged for 20 min at 10,000 rpm (Beckman Coulter Airfuge Air-Driven Ultracentrifuge, Indianapolis, IN, USA) and the supernatant containing non-adsorbed nucleic acid was used for SAM quantification. The adsorption efficiency (AE) was calculated using the method described above.

### 2.5. Physiochemical Characterization of Formulations

All formulations were characterized in terms of hydrodynamic size (Z-average diameter, the intensity weighted mean hydrodynamic size), polydispersity index (PDI) and zeta potential (ZP) by dynamic light scattering (DLS) in a Zetasizer Nano ZS (Malvern Panalytical, Malvern, UK) at 0.1–0.2 mg/mL at 25 °C in their respective buffer. 

### 2.6. RNase Protection Assay

To assess the ability of cationic formulations to protect SAM-RVG from RNase degradation, each SAM-RVG/formulation complex was exposed to 3.8 mAU of RNase A (Ambion, Austin, TX, USA) per microgram of SAM for 30 min at room temperature. RNase was inactivated by incubation with proteinase K (Novagen, Merck KGaA, Darmstadt, Germany) at 6.4 mAU/µg of SAM for 10 min at 55 °C. To extract the SAM from lipid formulations, 950 µL of ethanol were added to 50 µL of each sample, mixed and centrifuged at 12,000 rpm for 25 min. The resulting pellets were resuspended in 25 μL of DEPC-treated water, mixed with formaldehyde load dye (1:3 v/v), heated at 65 °C for 10 min and then cooled to room temperature on ice. SAM integrity was analyzed by denaturing gel electrophoresis. 0.5 μg of SAM per lane was loaded in a denatured 1% agarose gel in Northern Max 3-(N-morpholino) propane sulfonic acid (MOPS) running buffer, containing 0.1% of SYBR gold stain (Invitrogen, Carlsbad, CA, USA), and run at 100 V. Ambion Millennium marker was used as the molecular weight standard. Gel images were acquired in a Gel Doc EZ imager (Bio-Rad, Hercules, CA, USA).

### 2.7. Cell Toxicity Assay in Baby Hamster Kidney Cells

Baby hamster kidney cells (BHK) (obtained from American Type Culture Collection, ATCC) were cultured in complete DMEM (DMEM supplemented with 5% HI-FCS and 1% penicillin and streptomycin). Cells were grown for 2–3 days at 37 °C and 5% CO_2_. BHK cells were plated at a density of 5 × 10^4^ cells in 500 µL of DMEM either in the presence (5%) or absence of HI-FCS in each well of a 24-well plate and incubated for at least 6 hours to allow cell adhesion. Then, cells were incubated with serial dilutions of SAM-RVG-liposomes, NPs, SLNs and emulsions, starting from a cationic lipid concentration of 100 µg/mL (which corresponded to a SAM-RVG concentration of 6 µg/mL) through a 3-fold serial dilution. After 16 hours, cells were trypsinized, transferred in 96-well plates and stained with 100 µL of diluted Live/Dead Fixable Aqua Dead Cell Stain for 20 min at room temperature in the dark. After two washes with PBS, cells were resuspended in PBS and the percentage of Live/Dead^+^ cells with respect to untreated control was measured by flow cytometry. All flow cytometry data were collected on a FACSCANTO II (BD BioScience, San Jose, CA, USA) flow cytometer and analyzed using FlowJo software 7.6 (BD BioScience, San Jose, CA, USA).

### 2.8. Cellular Association Efficiency in Baby Hamster Kidney Cells 

BHK cells were seeded at a density of 5 × 10^4^ cells in 500 µL of DMEM either in the presence (5%) or absence of HI-FCS in each well of a 24-well plate and incubated for at least 6 hours to allow cell adhesion. To track cell uptake, SAM-RVG particles were co-formulated with the lipophilic fluorescent dye DilC (0.2% mol) added in the lipid mixture prior to microfluidics mixing or emulsification. Formulations were then added at 11 μg/mL of cationic lipid (which corresponded to 0.7 μg/mL of SAM-RVG) and incubated for 16 hours at 37 °C and 5% CO_2_. As a positive control, BHK were also treated with Lipofectamine2000 (LF2000) at 5 µL/well following the manufacturer’s instructions. Subsequently, cells were harvested from the well-plates, washed and resuspended in PBS supplemented with 5% HI-FCS, and the percentage of DilC^+^ cells with respect to untreated control was measured by flow cytometry in a FACSCANTO II (BD BioScience, San Jose, CA, USA). All data were analyzed in FLowJo software 7.6 (BD BioScience, San Jose, CA, USA).

### 2.9. In Vitro Potency of SAM-RVG Formulations

BHK cells (5 × 10^4^ cells/well) were plated as previously described. After 6–8 hours, cells were incubated for 16 hours with SAM-RVG liposomes, NPs, SLNs and emulsions at SAM-RVG concentrations of 400, 200 and 100 ng/mL (corresponding to 6, 3 and 1.5 μg/mL of cationic lipid, respectively). LF2000 was used as a positive control, as mentioned above. Then, cells were trypsinized and transferred to 96-well plates, washed twice with PBS, fixed and permeabilized with Cytofix-Cytoperm solution containing 4% paraformaldehyde (PFA). After incubation, cells were washed twice with PBS and stained with primary antibody dilution (Anti-RVG Ab, clone 24-3F-10) for 1 hour at room temperature. Then, after washing, cells were incubated with secondary antibody dilution (Anti Mouse IgG2a) for 1 hour at room temperature in the dark. Finally, cells were washed and resuspended in PBS for flow cytometry in a FACSCANTO II (BD BioScience, San Jose, CA, USA) flow cytometer. The percentage of RVG^+^ formulations was calculated with respect to untreated control cells. All data were analyzed in FLowJo software 7.6 (BD BioScience, San Jose, CA, USA).

### 2.10. Evaluation of Immune Responses in Vivo of Different Selected Adjuvants and Their Associated Antigen

#### 2.10.1. Determination of Antigen-Specific Serum Antibody Titers by Enzyme-Linked Immunosorbent Assay (ELISA)

All animal studies were ethically reviewed and carried out in accordance with European Directive 2010/63/EEC and the GSK policy on the Care, Welfare and Treatment of Animals. Animals were placed in cages with an individually ventilated caging system and given a standard mouse diet ad-libitum. Groups of 10 female BALB/c mice (Charles River, Wilmington, MA, USA) aged 6–8 weeks and weighing about 20–25 g received either 1.5 or 0.15 μg of SAM-RVG encapsulating DOTAP liposomes, DDA liposomes and DOTAP NPs, or adsorbing CNE56 at days 0 and 28 in 50 μL in the left quadriceps. Similarly, 50 μL of Rabipur (which corresponded to 1/20 of clinical dose) was administered via i.m. injection to control groups. Serum samples from individual mice were collected on days 0, 27 and 43 after the first vaccination. Within this study, SAM alone was not tested. Previously published studies have confirmed that there were no antigen-specific humoral or cellular responses induced by unformulated SAM [40]. Similarly, Magini et al. immunized mice either with SAM NP or with SAM M1 (two influenza antigens), and no cross-reactive cellular immune responses were observed [18]. Thus, based on this, SAM alone was not tested to reduce the animal number and be compliant with 3Rs requirements.

RVG-specific IgG titers were determined by ELISA using a commercially available kit (PLATELIA RABIES II Kit Ad Usum Veterinarium). The assay was performed following the manufacturer’s instructions. Briefly, each sample was pre-diluted 1:100 in sample buffer and 100 μL was incubated in a microplate well sensitized with the rabies virus glycoprotein for 60 ± 5 min at 37 ± 1 °C. A negative (R3) and two positive controls (R4a and b) were tested in each run. The positive controls were calibrated against the World Health Organization (WHO) international standard for rabies immunoglobulin. R4b (4 equivalent units (EU)/mL) was used to establish a reference curve after successive two-fold serial dilutions (S5 = 2 EU/mL, S4 = 1 EU/mL, S3 = 0.5 EU/mL, S2 = 0.25 EU/mL, S1 = 0.125 EU/mL). After three washing cycles with 1× washing solution by the use of a microplate washer (type PW41 or PW40, Bio-Rad, Hercules, CA, USA), horseradish peroxidase-conjugated protein A (10× concentrated, to be diluted in 1× washing solution prior to use) was incubated for 1 h ± 5 min at 37 ± 2 °C in a microplate incubator (type IPS, Bio-Rad, Hercules, CA, USA). Following five washes, the linked peroxidase conjugate was visualized with 3,3’,5,5’-tetramethylbenzidine incubated for 30 ± 5 min at room temperature. The enzyme reaction was stopped by addition of 1 N sulfuric acid solution. Absorbance was measured at 450–620 nm with the use of a microplate reader (type PR3100, Bio-Rad Hercules, CA, USA) with a specific rabies program. The dose-optical density (OD) response curve allowed the determination of the titer of each serum. Sera titers were expressed in equivalent unit per mL (EU/mL).

#### 2.10.2. Intracellular Cytokine Staining (ICS) in Splenocytes

Spleens from three randomly selected immunized mice were removed at day 43 after the first vaccination. To assess antigen-specific T-cell responses, single splenocyte suspensions were prepared. In essence, spleens were homogenized in cold HBSS and filtered through 70 μm cell strainers, then washed with HBSS. Samples were then incubated with RBC lysis buffer (2 mL) at 4 °C for 2 min, then resuspended in complete RPMI (cRPMI) and filtered through cell strainers. Cells were counted in a Vi-CELL XR cell counter (Beckman Coulter, Brea, CA, USA) and plated at 15 × 10^6^ cells/mL with anti-CD28 mAb at a final concentration of 2 μg/mL and anti-CD107a FITC (5 μg/mL). As a positive control, cells were also pre-incubated for 2 hours at 37 °C with anti-CD3 mAb (1 μg/mL). Moreover, as ex vivo restimulation, cells were stimulated for 4 hours with RVG peptide pool G1/G2/G3 (consisting on 15-mers with 11 amino acid overlaps) at 2.5 μg/mL. Brefeldin A (5 μg/mL) was added to each condition for the last 4 hours. For flow cytometry analysis, cells were then stained with Live/Dead Near InfraRed, fixed and permeabilized with Cytofix/Cytoperm, and then incubated with anti-CD16/CD32 Fc block. T-cells were further stained with anti-CD3-APC, anti-CD4 BV510, anti-IFN-γ Brilliant Violet 785, anti-IL-2 PE-Cy5.5, anti-TNF Brilliant Violet 605 and anti-CD8 PE. Samples were acquired on a LRSII special order (BD Biosciences, San Jose, CA, USA) and analyzed using FlowJo software version 7.6 (BD Biosciences, San Jose, CA, USA). Frequencies of antigen-specific T-cells were calculated after subtracting the background measured in the corresponding negative control for each cytokine.

### 2.11. Statistical Analysis

Unless stated otherwise, the results were calculated as mean ± standard deviation (SD). One- way analysis of variance (ANOVA) followed by Tukey’s post hoc analysis was performed for comparison and significance was acknowledged for *p* values < 0.05. Analysis and interpretation were made using GraphPad Prism 8 (GraphPad Prism software, San Diego, CA, USA) and Microsoft Excel. 

## 3. Results

### 3.1. Evaluation of Physical Attributes of Liposomes, Solid Lipid Particles, Polymeric Particles and Emulsions Produced with SAM-RVG

A set of cationic nanoparticles (liposomes, SLNs, NPs and emulsions) either entrapping or adsorbing SAM encoding the rabies glycoprotein G (RVG) were prepared. Samples were analyzed according to their physiochemical properties. Criteria applied to screen formulations were based on previous work on LNP systems [41]. These criteria included: small particle size (with a target size range of 50–200 nm), monodisperse size distribution (target range, polydispersity index (PDI) < 0.3) and high loading efficacy (target range of encapsulation/adsorption efficiency (EE/AE)> 90%). In addition to this, zeta potential was measured to assess the impact of loading SAM into these systems.

The four delivery platforms (liposomes, SLNs, NPs and emulsions) exhibited different compositions, but all contained the same cationic lipid concentration (either DOTAP or DDA) and a summary of their physico-chemical characteristics is shown in Table 1 (a scheme showing the concept/structure of these nanoparticles and encapsulation/adsorption is shown in Supplementary Figure S1). When considering liposomes formulated without SAM-RVG, both DDA and DOTAP liposomes were similar in terms of physico-chemical attributes: they were approximately 40–50 nm in size, with PDI < 0.3 and highly cationic in nature (55–60 mV for DOTAP and 40–50mV for DDA formulations) (Table 1). When SAM was mixed with these preformed liposomes (referred to as adsorbed SAM), the particle sizes significantly (*p* < 0.05) increased (100–145 nm for DOTAP formulations and 170–200 nm DDA formulations) and the zeta potential reduced due to the high (95%–99%) adsorption of the SAM-RVG (Table 1). When the SAM-RVG was entrapped within the liposomes, the impact on particle size was less notable for DOTAP liposomes (80–90 nm), which were cationic in nature (25–30 mV) and with high SAM-RVG loading (Table 1). However, entrapment of SAM-RVG within DDA liposomes produced particles of similar size to those where the SAM-RVG was adsorbed (Table 1). Overall, high loading (>95%) was achieved, irrespective of the cationic lipid employed and the manufacturing method (Table 1).

In the case of the SLNs, when formulated in the absence of SAM-RVG, the particles were 65–75 nm, with a low PDI (<0.3), and cationic in nature, irrespective of the cationic lipid used (Table 1). When these cationic SLNs were mixed with SAM-RVG, a significant (*p* < 0.05) increase in particle size was again seen in combination with a reduction in zeta potential and high SAM-RVG loading (>95%; Table 1). As for the liposomes, the DDA-based SLNs were larger than their DOTAP counterparts (120 nm for DOTAP compared to 200 nm for DDA-based SLNs; Table 1). When SLNs were formulated to entrap SAM-RVG, only SLNs containing DOTAP could be prepared as notable aggregation was observed during DDA-based SLNs entrapping SAM-RVG manufacturing. Polymeric nanoparticles formulated with either DOTAP or DDA were 40–60 nm in size, with a low PDI and a high zeta potential (Table 1). When these nanoparticles were mixed with SAM-RVG, the DOTAP-based nanoparticles precipitated and the DDA-based nanoparticles significantly (*p* < 0.05) increased in diameter, up to 260–270 nm (Table 1). In contrast, when the polymeric nanoparticles were formulated to entrap SAM-RVG, only those containing DOTAP could be prepared with vesicle sizes of approximately 200 nm (PDI 0.23), cationic zeta potential (20–30 mV) and high (>95%) SAM-RVG incorporation. Finally, prior to addition of SAM-RVG to emulsions, globule sizes were 140–160 nm for DOTAP-based emulsions and 170–210 nm for DDA-based emulsions (Table 1). Both were cationic in nature (30–40 mV) and with the addition of SAM-RVG, high adsorption was noted (>95%) combined with an increase in globule size and a drop in zeta potential (Table 1). Given the construct of emulsions, SAM-RVG entrapment could not be considered. 

Overall, prior to the addition of SAM-RVG, the choice of cationic lipid used (DOTAP versus DDA) did not notably impact on particle size and all systems were highly cationic (Table 1). On the addition of SAM-RVG (either entrapped or adsorbed), all formulations had high loading and particle sizes increased, and generally, DDA-based formulations were larger in size (Table 1). When liposomes, SLNs, NPs and emulsions were tested for their ability to protect their associated SAM-RVG, formulations showed variable protection with no clear trend in terms of either delivery system or DOTAP versus DDA (Supplementary Figure S2).

### 3.2. Cell Association of SAM-RVG-Loaded Liposomes, SLNs, NPs and Emulsions in BHK Cell Line

**Figure 1:**
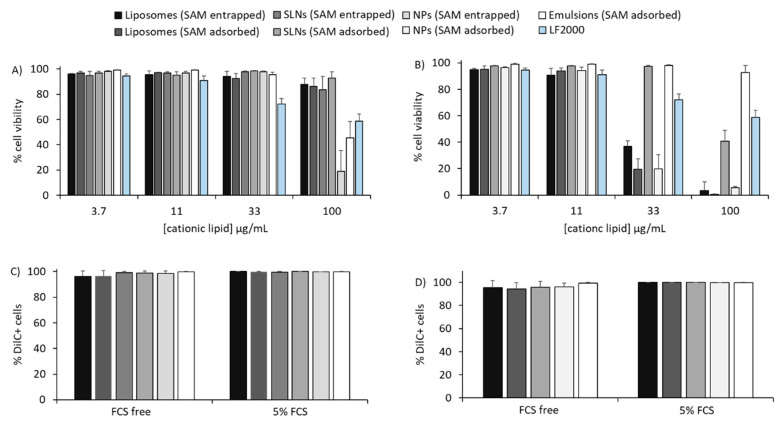
Cell viability and cell association of delivery platforms with baby hamster kidney (BHK) cells. Cytotoxicity (**A**,**B**) and cell association (**C**,**D**) in BHK cell of DOTAP-based (**A**,**C**) and DDA-based (**B**,**D**) liposomes, SLNs, NPs and emulsions. Lipofectamine 2000 (LF2000) was used as positive control. For cell viability, cationic lipid concentrations of 3.7 to 100 µg/mL (N:P ratio of 8:1) were tested. For cell association, the results represent percentage of 1,1’-dioctadecyl-3,3,3’,3’-tetramethylindocarbocyanine perchlorate (DilC)-positive BHK cells after 16 hours of incubation with 11 µg/mL of DOTAP-based (C) and DDA (D) formulations in either 5% fetal calf serum (FCS) or FCS-free media. Results are represented as mean ± SD of 3 independent experiments.

Based on these initial studies, the various formulations outlined in Table 1 progressed into in vitro studies. Initially, the cytotoxicity of all formulations in BHK cells was quantified after 16 hours by the amine reactive dye Live/Dead to identify the sub-toxic concentration to work at. Generally, DOTAP formulations were less toxic compared to DDA formulations, with cells remaining viable at concentrations up to 33 µg/mL for DOTAP-based delivery systems compared to 11 µg/mL for DDA-based delivery systems (Figure 1A,B). The exception to this was the emulsions formulations, with cells having high viability when treated with up to 100 µg/mL for both the DOTAP and DDA formulations (Figure 1). Based on these results, delivery system concentrations of 11 µg/mL or less were used within the cell studies. To investigate the impact of cationic lipid choice on cellular association, fluorescently labelled DOTAP- (Figure 1C) and DDA (Figure 1D)-based formulations were prepared. Initially, all delivery systems were incubated for 16 hours in cells in FCS-free medium or complete medium (i.e., FCS-free or medium containing 5% FCS, respectively; Figure 1C,D). By expressing particle cellular association as percentage of DilC^+^ cells, there was no significant difference between the various DOTAP- and DDA-based delivery systems (Figure 1C,D); thus, despite differences in physico-chemical properties, the different cationic lipid used and the different cells’ growing conditions (+/− 5% FCS), formulations were associated with fibroblasts to the same extent, with an average percentage of DilC-positive cells of around 95% (Figure 1C,D).

**Figure 2:**
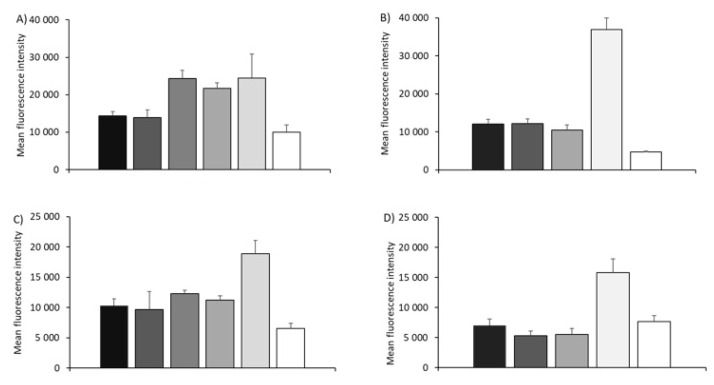
Cell association of formulations in BHK cell line expressed as mean fluorescence intensity (MFI). Mean fluorescence intensity of BHK cells after 16 h incubation with DOTAP-based (**A**,**C**) and DDA-based (**B**,**D**) liposomes, SLNs, NPs and emulsions in either 5% FCS (**A**,**B**) or FCS-free media (**C**,**D**). Results are represented as mean ± SD of 3 independent experiments.

However, differences in association efficiency between the formulations were evident when analyzing mean fluorescence intensity values (MFI) (Figure 2). The mean fluorescence intensity measured by flow cytometry is equivalent to the amount of fluorescent dye associated with BHK, which is directly proportional to the product of the number of particles by particles’ fluorescence intensity. Figure 2A shows that the MFI of DOTAP SLNs and NPs was significantly higher (*p* < 0.05) compared to the liposome- and emulsion-based formulations: SLNs and NPs MFI was >24,300, whilst the DOTAP-based liposome and emulsion systems had MFI < 14,300 (Figure 2A). With the DDA-based delivery systems, only the NPs showed significantly (*p* < 0.05) higher levels compared to the other formulations (Figure 2B) and in general, moving from DOTAP to DDA reduced the overall cell uptake based on MFI (Figure 2A,B). When these studies were conducted in FCS-free conditions (Figure 2C,D), whilst absolute values were lower compared to those measured in complete medium, a similar trend was noted across the delivery systems and comparing the two different cationic lipids (Figure 2).

### 3.3. In Vitro Potency Assay with SAM-RVG Formulations 

**Figure 3:**
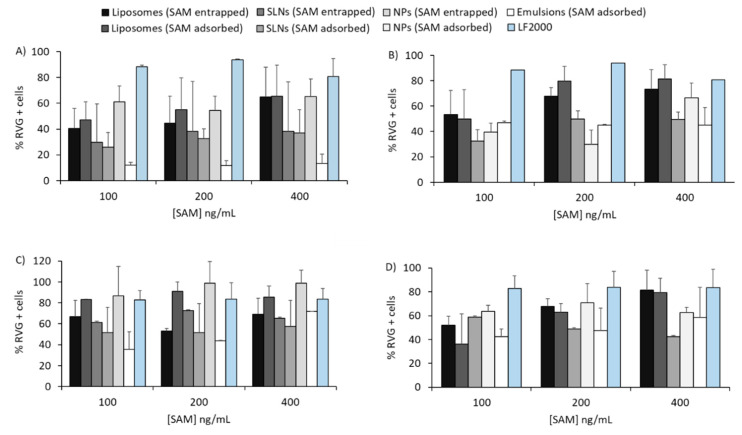
In vitro potency (IVP) in BHK cell line. IVP in BHK in either 5% FCS (**A**,**B**) or FCS-free (**C**,**D**) medium of DOTAP-based (**A**,**C**) and DDA-based (**B**,**D**) formulations prepared with SAM-RVG at different concentrations. Lipofectamine 2000 (LF2000) was used as a positive control. Results are represented as mean ± SD of 3 independent experiments.

Figure 3 represents the percentage of RVG-positive cells after 16 h incubation of SAM-RVG-loaded DOTAP (Figure 3A,C) or DDA (Figure 3B,D) particles at different antigen concentrations in complete medium. Overall, the transfection efficiency was SAM dose-independent: within the concentration range tested, increasing SAM-RVG content did not result in enhanced antigen expression. However, although all delivery systems tested did transfect fibroblasts, the potency of the transfection varied. In complete medium, with the DOTAP-based delivery systems, at SAM-RVG of 100 ng/mL, NPs and liposomes (with SAM-RVG entrapped or adsorbed) transfected between 40% and 60% of BHK cells, whilst for the rest of the delivery systems, the percentage of RVG^+^ cells was <30% (Figure 3A). Absolute values of RVG^+^ cells were not significantly different between the liposomes, SLNs and NPs at lower concentrations (Figure 3A); however, at SAM-RVG concentrations of 400 ng/mL, NPs and liposomes showed transfection efficiencies comparable to Lipofectamine 2000 (LF2000), whilst the percentage of RVG^+^ cells after treatment with SLNs and emulsion were lower (<50% %RVG^+^) compared to LF2000 (Figure 3A). A similar pattern was observed with DDA-based formulations (Figure 3B). Due to a possible inhibitory effect by serum proteins on gene delivery, in vitro potency (IVP) testing was also performed in the absence of serum in culture (Figure 3C,D). Particles-mediated antigen expression in serum-free medium followed a similar trend to that observed in complete medium conditions, but overall, the percentage of transfected cells increased in FCS-free conditions (Figure 3C,D). 

### 3.4. Immunogenicity of Different Vaccine Candidates Encoding RVG after Intramuscular Injection 

Immunogenicity of SAM-RVG vaccines was performed in BALB/c mice. Candidates were selected based on a down-selection strategy with those showing appropriate physical attributes progressing in vitro, and then based on in vitro potency (IVP), three delivery systems progressed to the in vivo study: DOTAP liposomes, DDA liposomes and DOTAP NPs, as summarized in Table 2. These three candidates were compared to Rabipur, a commercial inactivated rabies virus vaccine, and to CNE56. The SAM-RVG dose range tested was based on previous findings on LNP systems used for SAM delivery in mice [10]. Two different antigen doses were used to evaluate any correlation between SAM dosing and immune response.

**Figure 4:**
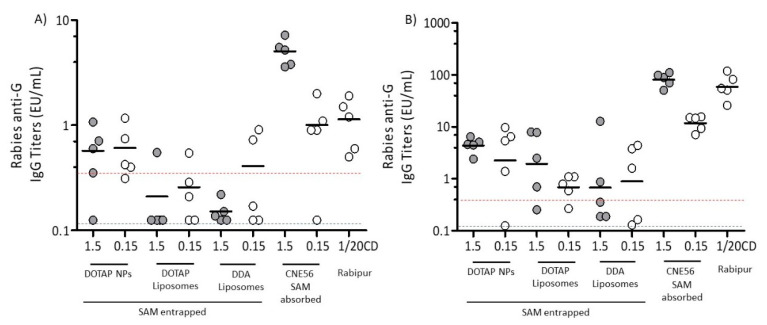
Immunogenicity of SAM-RVG vaccine delivered by different cationic carriers. Groups of ten BALB/c mice were immunized i.m. on days 0 and 28 with either 1.5 or 0.15 μg of self-amplifying RNA encoding the rabies virus G protein formulated in DOTAP polymeric nanoparticles (NPs), DOTAP Liposomes or DDA Liposomes, and compared to the commercial vaccine Rabipur (1/20 of human dose). GSK trademark CNE56 was used as a positive control. Specific IgG titers were measured by enzyme-linked immunosorbent assay (ELISA). For each group, there were 5 samples, each representing data from pools of two mice (depicted as circles), and the geometric mean titers (GMTs) are solid lines. Sera were collected and analyzed (**A**) 4 weeks after the first immunization and (**B**) 2 weeks after the second immunization. Titers < 0.125 EU/mL (dotted blue line) were below the limit of detection, while titers > 0.5 EU/mL (dotted red line) were an indication of protection. Intergroup comparison was analyzed using the one-way analysis of variance (ANOVA) test (Dunnett’s multiple comparison test).

Figure 4 shows the immunogenicity of SAM-RVG vaccines induced by the selected delivery systems at different time points. A study conducted in collaboration with WHO reported a concordance of 95% between IgG titers from this ELISA method and the rapid fluorescent focus inhibition test (RFFIT) test, a reference method for detection of protective levels of rabies neutralizing antibodies in sera [42,43]. Therefore, IgG titers measured within the current study can be correlated with functional neutralizing antibodies. Figure 4A shows the results four weeks after the first injection. It can be seen that, whilst mice which received CNE56 with adsorbed SAM showed dose-dependent responses, with all three of the other formulations, the lower dose (0.15 μg/dose) gave equivalent or even improved responses in terms of both the number of responders and the geometric mean titers (GMT) compared to the higher dose (1.5 μg/dose, Figure 4A). With mice that received NPs, the low dose of SAM (0.15 μg/dose) promoted antibody titers, not significantly different to those induced by Rabipur and CNE56 with a SAM dose of 0.15 μg. Moreover, at both SAM concentrations, NPs GMT was above 0.5 EU/mL, which is considered a positive protective antibody response to rabies vaccination according to WHO [44]. DOTAP NPs were also more effective than both liposome formulations (Figure 4A). When comparing between the liposome formulations, there was no significant difference in terms of cationic lipid used within the formulation (DOTAP versus DDA; Figure 4A). 

At day 43 (two weeks after the second immunization), all formulations increased in titers (Figure 4B) and generally, the pattern in terms of responses matched those seen four weeks after the first injection (Figure 4A). Of the three novel formulations, again, NPs generally elicited higher and more consistent glycoprotein G-specific IgG levels than both liposome formulations (Figure 4B). Comparing between the liposomes, whilst both formulations elicited comparable antibody titers at a 0.15 µg/dose of SAM, at the higher dose, DOTAP liposomes were more potent than DDA liposomes (Figure 4B) and boosted glycoprotein G-specific titers approximately 3-fold (GMT 2.25) relative to DDA liposomes (GMT 0.67) (Figure 4B). From these results, out of the novel formulations tested, NPs gave the most robust responses and were as potent as CNE56 at SAM concentrations of 0.15 µg/mL both prior to and post booster injection; however, CNE56 at SAM concentrations of 1.5 µg/mL gave significantly high responses (Figure 4). There was also no significant difference between NPs and the commercial vaccine Rabipur after the first injection, but after the second immunization, Rabipur was significantly (*p* < 0.05) more potent than the NP and liposome formulations (Figure 4). 

**Figure 5:**
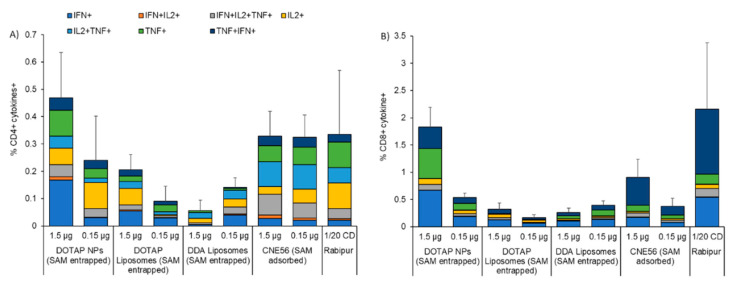
Percentages of antigen-specific CD4^+^ or CD8^+^ T-cells. Splenic (**A**) CD4^+^ T cells, and (**B**) CD8^+^ T-cells 2 weeks after two intramuscular immunizations spaced 4 weeks apart in BALB/c mice. Mice were immunized with either 1.5 or 0.15 μg/dose of self-amplifying RNA expressing rabies G glycoprotein adjuvanted with either polymeric nanoparticles (NPs), DOTAP liposomes or DDA liposomes, and spleens from 3 mice randomly selected out of the group were tested. Candidates were compared with the commercial vaccine Rabipur (1/20 of human dose) and GSK trademark CNE56 as positive controls. Splenocytes were stimulated with rabies G1/G2/G3 peptide, stained for intra-cellular cytokines, and analyzed by flow cytometry. The color code indicates the different combinations of cytokine produced by the respective cells. Unstimulated cells were used as control. Refer to Figure S3 in the Supplemental Material for the gating strategy.

After two immunizations, all animals had measurable RVG-specific CD4^+^ and CD8^+^ T-cell responses (Figure 5). Although not statistically significant, NPs at 1.5 µg of SAM induced the highest frequency of RVG-specific CD4^+^ T-cells, compared to all the other formulations, including Rabipur and CNE56 (0.46% compared to 0.35% and 0.33%, respectively; Figure 5A). At 0.15 µg of SAM, the RVG-specific CD4^+^ T-cells induced by SAM encapsulating NPs decreased to 0.25%. A similar dose-response was observed for the DOTAP liposome formulation, though the RVG-specific CD4^+^ T-cells’ frequencies were lower (0.21 and 0.1 at 1.5 and 0.15 µg, respectively). However, this was not evident for the DDA formulation (between 0.8% and 1.5% regardless of the SAM dose; Figure 5A). With respect to the cell phenotype, the quality of the CD4^+^ T cell response was similar for all the tested formulations: overall, CD4^+^ T-cells were poly-functional, expressing IFN-γ in combination with TNF and/or IL-2, a typical feature of mature effector CD4^+^ T-cells (Figure 5A). As expected, a combination of Th0 (IL-2^+^/TNF-α^+^, TNF-α^+^ and IL-2^+^) and Th1 (IFN-γ^+^/IL-2^+^/TNF-α^+^ and combinations) phenotype was observed in CD4^+^ T-cells two weeks after the second immunization (Figure 5A). Regarding CD8^+^ T-cell responses, again, NPs induced the highest frequency of RVG-specific CD8^+^ T-cells in a dose-dependent manner (2.6% and 0.55% at 1.5 and 0.15 µg, respectively; Figure 5B). NPs appeared to be more potent than CNE56 and as potent as Rabipur, whilst the DOTAP and DDA liposome formulations were not efficient in inducing CD8^+^ T-cells (Figure 5B). Comparing NPs and Rabipur-vaccinated mice, the quality of the response was slightly different, with more poly-functional CD8^+^ T-cells in the group of Rabipur-vaccinated mice, representing mature effector antigen-specific CD8^+^ T-cells (Figure 5B). However, it might be worth noting that the aforementioned observations were qualitative and due to the low number of animals analyzed, it was not possible to draw any statistical conclusions. Generally, NPs at SAM concentrations of 1.5 µg/mL were significantly more potent than both liposomes in inducing both cytokines producing CD4^+^ and CD8^+^ T-cells (*p* < 0.05) and as potent as both CNE56 and Rabipur.

**Figure 6:**
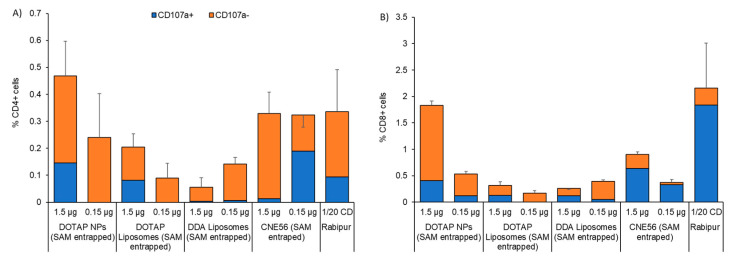
Percentages of cytotoxic CD4^+^ or CD8^+^ T-cells. The induction of rabies-specific CD4^+^ or CD8^+^ T-cells by either 1.5 or 0.15 μg/dose of SAM encapsulating nanoparticles (NPs), DOTAP liposomes and DDA liposomes was characterized 2 weeks after the second immunization. Spleens from 3 mice were randomly selected from each group. Candidates were compared with the commercial vaccine Rabipur (1/20 of human dose) and GSK trademark CNE56 as positive controls. Surface expression of CD107a on splenocytes stimulated in vitro with rabies G1/G2/G3 peptide was assessed by flow cytometry. Data show the frequency of cytokine-secreting (**A**) CD4^+^ or (**B**) CD8^+^ T-cells that express (blue bars) or do not express (orange bars) CD107a. Unstimulated cells were used as control. Refer to Figure S3 in the Supplemental Material for the gating strategy.

The effector phenotype of CD4^+^ and CD8^+^ T-cells was further evaluated by quantifying the surface expression of CD107a, a marker of degranulation which whose expression correlates with the in vivo cytotoxicity of T-cells [45] (Figure 6). After two immunizations with SAM-RVG at two different doses in combination with NPs or liposomes, the majority of RVG-specific CD4^+^ T-cells were CD107a- (Figure 6A). Similar results were obtained for CNE56 and the commercial vaccine Rabipur. This suggested that SAM formulations did not induce cytotoxic CD4^+^ T-cells (Figure 6A). However, considering specific CD8^+^ T-cells, candidates were less potent than Rabipur in inducing cytotoxic T-cells, in line with the phenotype observed (less polyfunctional CD8^+^ T-cells; Figure 6B).

## 4. Discussion

SAM vaccines offer strong potential compared with conventional vaccines as they can be produced by a simple, synthetic and cell-free process. Furthermore, SAM vaccines can encode not only the antigen but also the viral replication machinery that enables intracellular mRNA amplification and protein expression [46]. Co-formulation of SAM and lipid nanoparticles (LNPs) further supports increased vaccine potency, as LNPs augment in vivo stability by preserving the nucleic acid from enzymatic degradation and facilitating accumulation within cell cytosol [10]. However, other formulation platforms may also offer similar advantages in terms of SAM delivery. Cationic liposomes were the first to be used extensively for the delivery of nucleic acid therapeutics, such as pDNA [23,47] and siRNA [48,49]. Since the early works conducted in the 1980s [50,51], the number of studies aiming to evaluate the potential of liposomes for gene therapy have markedly increased. To date, cationic liposomes have been applied in more than 100 clinical trials, mainly for cancer therapy and monogenic diseases like cystic fibrosis [52]. Nanoemulsions have also been described as a means to improve transfection of nucleic acids and they have been evaluated in humans for a range of vaccine candidates, including those targeting herpes simplex virus (HSV), human immunodeficiency virus (HIV), hepatitis C virus (HCV) and cytomegalovirus (CMV) [53,54,55]. More recently, a cationic version of the squalene-based emulsion-MF59, which contains the cationic lipid DOTAP- has been used to potentiate the immune response of a SAM in both rabbits and non-human primates (NHP) [39], and recently entered a phase I clinical trial. Furthermore, the combination of DNA/mRNA with polymeric nanoparticles was successfully employed in gene therapy [56]. Polyethyleneimine (PEI) is one of the most prominently used cationic polymers to formulate nanoparticles for nucleic acid delivery. For example, it was observed that transfection of the HPV16 E7 gene with PEI-based nanoparticles was efficient in COS-7 cells, but toxicity was observed in vivo [57]. Therefore, more biodegradable polymers such as poly(lactic-co-glycolic acid)(PLGA) or polylactic acid (PLA) are preferred, as they are more biocompatible, and their degradation rate can be tuned to control the release of nucleic acid into the cytosol. Currently, most of the NPs-based RNA delivery systems are approved for clinical evaluation in cancer therapy, virus infection, cystic fibrosis and hypercholesterolemia [29]. In contrast to the aforementioned delivery systems, SLNs are less widely explored [34,58]; although promising, their main application remains as solubilizing agents for the delivery of poorly soluble drugs [59] or in the cosmetic and food industry [60], and only limited studies report their use as vaccine adjuvants, especially in combination with nucleic acids [61,62].

Within this study, we have investigated a set of cationic formulations for SAM vaccine delivery: liposomes, NPs, SLNs and emulsions. These alternative non-viral carriers were considered as a potentially more cost-effective strategy for in vivo delivery of SAM. To manufacture these systems, we adopted either microfluidics or microfluidization, which supported production of narrow-size and homogeneous empty or SAM-encapsulating/adsorbing particles. All formulations included a cationic lipid (either DOTAP or DDA), to produce cationic nanoparticles. Changing from DOTAP to DDA produced larger particles whilst still maintaining comparable surface charge values. This size modification of DDA-based carriers may be due to the intrinsic rigidity of particles formulated with this cationic lipid [63,64]. The strategy of using positively charged lipids for nucleic acid delivery is widely accepted, as they enhance antigen loading owing to charge-to-charge interactions with nucleic acid [65] and promote binding with the negatively charged cell membrane, thus facilitating cell uptake [66,67]. Furthermore, the combination of cationic and anionic lipids favors the formation of non-bilayer structures that enhance endosomal escape and cargo release within the cytosol [68]. Several publications suggest that a cationic charge is a crucial factor for retention of the vaccine at the injection site, thus prolonging antigen presentation to the innate immune cells [69]. The increased permanency of SAM-loaded particles at the injection site might be of interest as it was reported that internalization of mRNA vaccines occurs primarily by nonimmune cells at the injection site and the antigen is expressed mainly by muscle cells, fibroblast and keratinocytes [70]. However, some publications hypothesized that a strong depot effect might not be required for these types of formulations, thus the role of SAM deposition at the injection site remains unclear [37]. Despite differences in physico-chemical attributes, all formulations were able to protect SAM-RVG from ribonuclease degradation. As previously reported, protection from enzymatic degradation significantly increased levels of reporter gene expression compared to naked RNA and lowered variability of expression between animals [10,53]. 

Cationic particles readily associated with BHK cells in vitro, with approximately 95% Dilc^+^ cells regardless of the presence or absence of serum proteins. Although the uptake mechanism of SAM-loaded particles was not investigated in the present study, we speculate that the association pathway of non-ionizable cationic particles did not involve the endogenous ligand Apolipoprotein E (ApoE). Accordingly, literature reported that ApoE plays a major role as an endogenous targeting ligand for ionizable LNPs, but not cationic LNPs [71]. The interaction of ionizable LNPs siRNA with ApoE was crucial for efficient hepatocyte gene silencing after i.v. administration [71], whereas this mechanism might not be essential for the delivery of SAM administered i.m. [71]. In vitro potency (IVP) experiments showed that DOTAP polymeric nanoparticles and both DDA and DOTAP liposomes induced the most consistent antigen expression in BHK cells. The improved ability of either DOTAP or DDA liposomes to elicit antigen expression might be helped by the presence of the fusogenic phospholipid DOPE in the liposome formulations. The cone-like shape lipid geometry can stabilize the non-bilayer hexagonal (H_II_) phase, which is found in transitional structures during membrane fusion and/or bilayer disruption [72,73]. On the other hand, the potency of polymeric NPs to transfect fibroblasts might also be attributed to the presence of the biodegradable polymer PLGA in the formulation [74]. The mechanism of the enhanced therapeutic effect given by PLGA has been elucidated by Panyam and co-workers [75]. They postulated that at the acidic pH of endo-lysosomes (pH between 4 and 5) [76], the lactic and glycolic acid monomers of PLGA become protonated, hence interacting with the negatively charged membrane, leading to their escape into cytoplasmic compartment. The mechanism of escape proposed within the cited paper was attributed to localized destabilization of the endo-lysosomal membrane at the point of contact with PLGA NP, followed by extrusion of the NP through the membrane [75]. 

Our immunogenicity studies in mice showed that, after a single immunization, NPs induced IgG titers comparable to the commercial vaccine Rabipur and CNE56. The strong correlation between the aforementioned IgG titers and the RFFIT test established by WHO [42,43] suggested that immunoglobulin titers measured within this paper could be correlated with functional neutralizing antibodies. NPs GMT was above the level of protective antibody response to rabies vaccination reported by WHO [44]. After the boost, NPs at 0.15 μg/dose of SAM were as potent as CNE56 to elicit antibody titers in vivo. PLGA nanoparticles for nucleic acid delivery have attracted strong interest as an alternative to the commonly used polycationic carriers that can be toxic and/or non-biodegradable [74]. PLGA has been licensed by the FDA and European Medicines Agency (EMA) for the delivery of therapeutics via subcutaneous, intradermal, intramuscular and mucosal routes [77,78]. PLGA has also been adopted in the manufacturing of biomedical devices like surgical sutures and bone implants [79]. To date, 15 PLGA-based products are marketed for clinical use as cancer vaccines [80]. In order to increase nucleic acid association, PLGA nano/microparticles are usually formulated with cationic materials such as DOTAP or cetyltrimethylammonium bromide (CTAB) [81]. Accordingly, PLGA/CTAB microparticles entered stage I clinical trials by Novartis for HIV-1 DNA vaccination [82]. In addition, PLGA-encapsulated DNA encoding human papillomavirus (HPV) antigen has been tested in phase II human clinical trials [83]. 

Despite promising results on antigen transfection in vitro, DOTAP and DDA liposomes were less potent than NPs to elicit a robust humoral response in mice and the variability among mice was high. The poor correlation between in vitro protein expression and in vivo immunogenicity was also reported by Blakney and co-workers, where different LNPs (based either on DDA, DOTAP or C12-200) showed different in vitro and in vivo antigen expression, but comparable antibody titers levels in vivo [38]. This was also seen elsewhere [37,84]. Interestingly, after the boost, DOTAP liposomes were more efficient in eliciting IgG titers compared to DDA counterparts. This fact could be explained by considering studies of Hassett and co-workers, who demonstrated a relationship between both immunogenicity and expression in vivo and particles size, with the best performing formulations being 75–95 nm [37]. As discussed previously, DOTAP liposomes were around 75 nm in size, while DDA particles diameter was around 200 nm. This finding might correlate with observed differences in IgG titers between DOTAP and DDA liposomes. DDA is commonly employed as a potent adjuvant which has previously been shown to enhance immunogenicity of experimental subunit vaccines [85,86,87]. It is widely accepted that DDA exerts a strong stimulatory effect by activating innate immunity, cell-mediated immunity and delayed-type hypersensitivity [88,89]. However, strong activation of the innate immune response may also offer drawbacks, as it could hamper the amplification of the RNA replicon and the expression of the encoded antigen, due to overexpression of type 1 IFN [90]. This would inevitably affect SAM efficacy in vivo. Moreover, it was observed that fully saturated lipids might not be suitable for mRNA and siRNA delivery, while lipids containing 2 to 3 unsaturated hydrocarbons assume a non-bilayer H_II_ structure more rapidly. These structures are known to destabilize endosomal membrane and facilitate RNA release in the cytosol [91]. 

The T-cell assay performed in splenocytes from mice that received the boost showed that frequencies of cytokine-producing CD4^+^ and CD8^+^ T cells induced by SAM-encapsulating NPs were comparable to those of Rabipur and CNE56. Moreover, NPs-induced CD4^+^ T-cells showed a Th1 phenotype, with several polyfunctional cells. SAM-NPs induced a similar phenotype in CD8^+^ T-cells to Rabipur, however, the quality of released cytokines was different. This finding correlated with the observed cytotoxic profile and was in line with what has been already documented in the literature [19,39,54,92]. Overall, results reported here showed differences in quantity rather than quality of cytokines proliferation induced by the SAM-loaded candidates tested, with NPs eliciting higher CD4^+^ and CD8^+^ cytokines release compared to both liposomes. This was consistent with differences in immunogenicity observed within this work. It has been seen that PLGA might play a role in directing the immune response. For instance, the loading of Hepatitis B core antigen into PLGA NPs induced a stronger cellular immune response as compared with Hepatitis B core antigen alone in a mouse model [93]. Moreover, a correlation between particle size and immunogenicity was evaluated previously, with NPs ranging from 200 to 600 nm associated with higher levels of IFN-γ production related to a Th1 response, whereas increasing particles size up to 2000 nm promoted IL-4 secretion related to a Th2 response [94].

## 5. Conclusions

The present study demonstrates that it is possible to effectively formulate and manufacture four different cationic formulations produced by two manufacturing methods: microfluidic mixer and microfluidization. Our data showed that the choice of delivery platform and method of SAM association (entrapment versus adsorption) impacts on the physico-chemical attributes. Prior to the addition of SAM, liposomes and nanoparticles were <65 nm, whilst emulsions were 150 nm. The addition of SAM increased particle size and lowered their zeta potential, especially when adsorbed to the DDA-based particles. Based on in vitro potency, liposomes (DOTAP or DDA) entrapping SAM and DOTAP nanoparticles entrapping SAM progressed to in vivo testing. However, the lack of correlation between in vitro antigen expression and in vivo immune response is noted, thus indicating that in vitro expression alone is insufficient to identify improved SAM vaccine formulations. In vivo, DOTAP NPs are effective in inducing robust immune responses in mice, with comparable potency to the commercial vaccine Rabipur and the benchmark CNE56. Generally, the DOTAP NPs formulation identified herein is a promising alternative synthetic delivery carrier to commonly used LNPs and has the potential to be further optimized for the delivery of a self-amplifying RNA vaccine platform. 

## Figures and Tables

**Table 1:** Physico-chemical properties of cationic lipid-based delivery systems. All formulations were prepared at 1 mg/mL final cationic lipid concentration. Results are represented as mean ± standard deviation (SD) of three independent batches of each formulation. DOTAP (1,2-dioleoyl-3-trimethylammonium-propane), DDA (dimethyldioctadecylammonium) NPs (polymeric nanoparticles), SLNs (solid lipid nanoparticles), size (Z-average diameter), ZP (zeta potential), PDI (polydispersity index), EE (encapsulation efficiency), AE (adsorption efficiency).

Formulation Type		DOTAP				DDA			
		Size (d.nm)	PDI	ZP (mV)	EE/AE (%)	Size (d.nm)	PDI	ZP (mV)	EE/AE (%)
Liposomes	No SAM	42 ± 4	0.25 ± 0.01	58 ± 3	-	39 ± 6	0.20 ± 0.03	45 ± 7	-
	Adsorbed	118 ± 25	0.36 ± 0.20	39 ± 5	97 ± 1	186 ± 15	0.16 ± 0.02	27 ± 4	99 ± 0.1
	Entrapped	85 ± 5	0.17 ± 0.02	27 ± 3	96 ± 1	196 ± 9	0.21 ± 0.02	43 ± 3	99 ± 0.2
SLNs	No SAM	64 ± 1	0.11 ± 0.01	30 ± 4	-	70 ± 6	0.26 ± 0.02	46 ± 4	-
	Adsorbed	120 ± 2	0.21 ± 0.02	15 ± 2	97 ± 1	201 ± 62	0.29 ± 0.04	26 ± 4	99 ± 0.1
	Entrapped	187 ± 17	0.14 ± 0.01	23 ± 1	98 ± 1	-	-	-	-
NPs	No SAM	39 ± 9	0.13 ± 0.07	49 ± 6	-	58 ± 3	0.07 ± 0.02	38 ± 5	-
	Adsorbed	-	-	-	-	267 ± 34	0.27 ± 0.05	26 ± 4	99 ± 0.1
	Entrapped	198 ± 6	0.23 ± 0.02	26 ± 6	98 ± 1	-	-	-	-
Emulsions	No SAM	150 ± 7	0.05 ± 0.01	38 ± 2	-	196 ± 24	0.19 ± 0.07	38 ± 3	-
	Adsorbed	182 ± 33	0.19 ± 0.01	35 ± 2	95 ± 2	209 ± 9	0.08 ± 0.03	35 ± 3	90 ± 0.5
	Entrapped	-	-	-	-	-	-	-	-

**Table 2:** Schematic representation of criteria applied to DOTAP- or DDA-based liposomes, solid lipid nanoparticles and emulsions to down-select formulations to progress to in vivo studies.

			Progression Selection Criteria	
Cationic Lipid	Delivery Platform	Method of Association	Progress to In Vitro Based on Physio-Chemical Attributes	Progress to In Vitro Based on In Vitro Efficacy
**DOTAP**	Liposomes	Adsorbed	✓	✕
		Entrapped	✓	✓
	SLNs	Adsorbed	✓	✕
		Entrapped	✓	✕
	NPs	Adsorbed	✕	✕
		Entrapped	✓	✓
	Emulsions	Adsorbed	✓	✕
**DDA**	Liposomes	Adsorbed	✓	✕
		Entrapped	✓	✓
	SLNs	Adsorbed	✓	✕
		Entrapped	✕	✕
	NPs	Adsorbed	✓	✕
		Entrapped	✕	✕
	Emulsions	Adsorbed	✓	✕

## Supplementary Materials

The following are available online at https://www.mdpi.com/2076-393X/8/2/212/s1, Figure S1: Schematic showing the concept of the four different delivery platforms, Figure S2: Denaturing RNA agarose gel electrophoresis showing protection of SAM-RVG from RNAse delivery platforms, Figure S3. Gating strategy and representative dot plots to evaluate the immune response elicited by different selected adjuvants and their associated antigen after i.m. injection in vivo.
