# Supplementary material for: Investigating the Impact of Delivery System Design on the Efficacy of Self-Amplifying RNA Vaccines
Source: Vaccines (Basel). 2020 May 8;8(2):212. doi: 10.3390/vaccines8020212 (PMC7348957; doi:10.3390/vaccines8020212)
Supplement: Supplementary file 1 [file vaccines-08-00212-s001.pdf]

Supplementary Figures

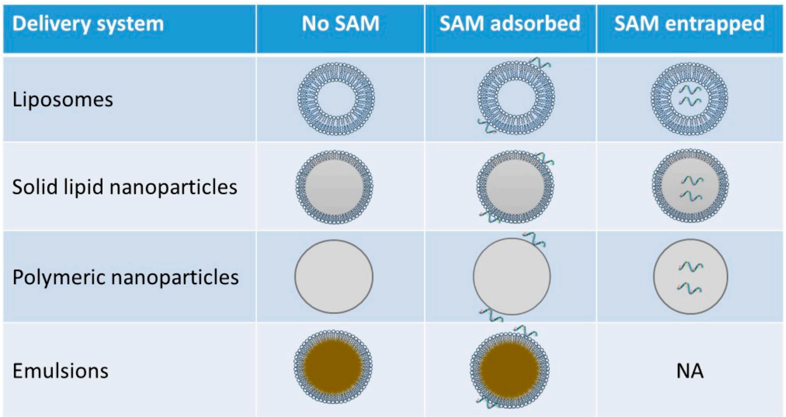

Figure S1. Schematic showing the concept of the four different delivery platforms. Each of these delivery systems were prepared containing a cationic lipid (DOTAP or DDA). These formulations were prepared without SAM, with SAM adsorbed or with SAM entrapped. To adsorb SAM on the surface, the SAM was added (8:1 mol/mol N:P) dropwise on into the suspensions of liposomes, SLNs, NPs and emulsions under mild stirring. SAM adsorbing formulations were allowed to complex at 4°C for at least 2 hours. To encapsulate SAM inside formulations, liposomes, SLNs and NPs were formulated with the addition of SAM (8:1 mol/mol N:P) in the aqueous phase prior to formulating the particles.

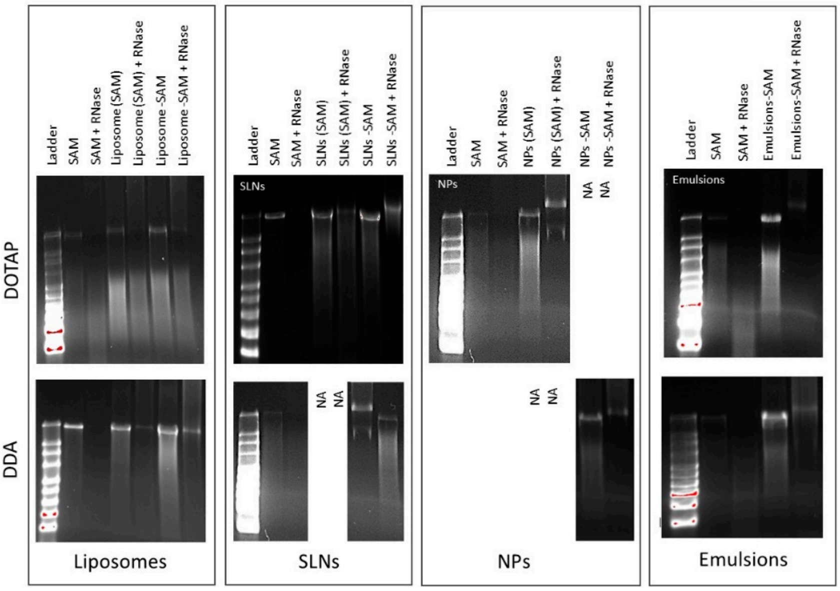

Figure S2. Denaturing RNA agarose gel electrophoresis showing protection of SAM-RVG from RNAse delivery platforms. Liposomes, SLNs, NPs and emulsions were prepared with either DOTAP or DDA as their cationic lipid component. These formulations either had entrapped ((SAM)) or adsorbed (-SAM) SAM-RVG and were then mixed with RNase. Molecular weight ladder (lane 1), SAM-RVG (lane 2), SAM-RVG after incubation with RNase (lane 3) were used as a control in all gels run. NA represents samples not tested due to initially phyisco-chemical instability.

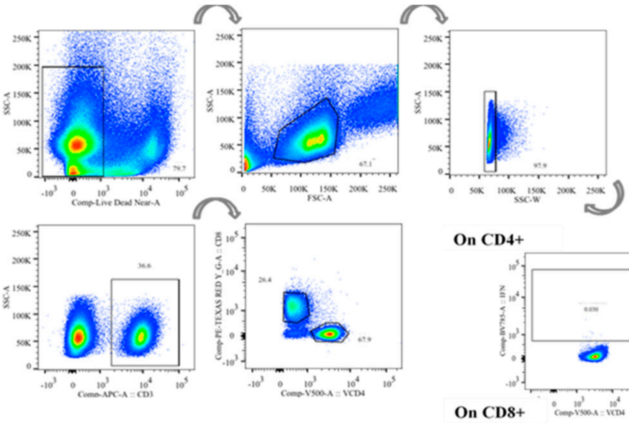

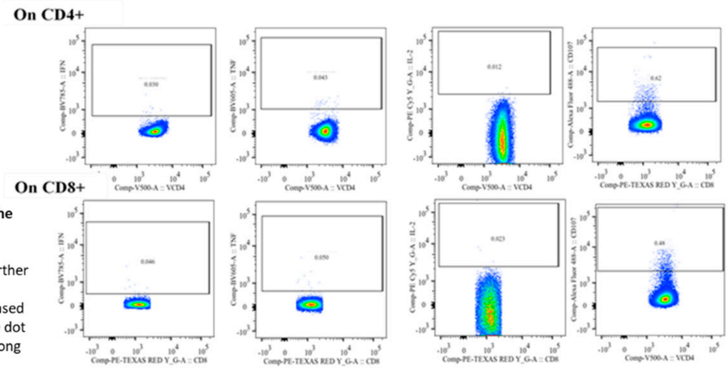

Figure S3. Gating strategy and representative dot plots to evaluate the immune response elicited by different selected adjuvants and their associated antigen after i.m. injection *in vivo*. Splenocytes were negatively selected based on dye exclusion, and lymphocytes were further identified based on morphology. CD3+ T cells were selected after discrimination of singlets and $CD4^{+}$ and $CD8^{+}$ T cells were identified based on CD4 and CD8 expression, respectively. Figure shows representative dot plots of cytokine+ (IFN-γ, IL-2, TNF-α| and CD107a) cells identified among the $CD4^{+}$ or $CD8^{+}$ subset.
